# Assessment of Computer Vision Syndrome and Personal Risk Factors among Employees of Commercial Bank of Ethiopia in Addis Ababa, Ethiopia

**DOI:** 10.1155/2021/6636907

**Published:** 2021-05-07

**Authors:** Haile Derbew, Ansha Nega, Worku Tefera, Tekie Zafu, Kenfe Tsehaye, Kebede Haile, Belsity Temesgen

**Affiliations:** ^1^Public Health Faculty, College of Health Sciences, Aksum University, Aksum, Ethiopia; ^2^Public Health Faculty, College of Health Sciences, Addis Ababa University, Addis Ababa, Ethiopia; ^3^Reproductive Health Faculty, College of Health Sciences, Debre Markos University, Debre Markos, Ethiopia

## Abstract

**Background:**

Computer vision syndrome (CVS) is an amalgam of visual symptoms caused by continued use of computers. Worldwide, up to 70 million workers are at risk for computer vision syndrome resulting in reduced productivity at work and reduced quality of life. Bank employees are among the risky workers with unknown magnitude of the syndrome. Therefore, the main aim of this study was to determine the prevalence of CVS and its associated personal factors among employees of Commercial Bank of Ethiopia.

**Methods:**

A total of three hundred and fifty-nine bank workers participated in the study between February and March 2018. A self-administered structured questionnaire was used to collect sociodemographic data, CVS symptoms, and its personal factors. Snellen chart tool was used to measure blurred vision. Data entry and analysis were performed via Epi Info™ 7 and Statistical Package for the Social Sciences (SPSS) version 21. Binary logistic regression and multivariable logistic regression were performed to assess the association and control the potential confounders.

**Result:**

The prevalence of computer vision syndrome in the last 12 months among the total study subjects, 359 (98% response rate), was 262 (74.6%) (95% confidence interval [CI] = 70.1, 79.5). Risk factors that could not be intervened with were sex (AOR: 1.8; 95% CI (1–3)) and age group (AOR: 3.11; 95% CI (1.2–8)). Causal factors that could be intervened with were use of electronic materials outside work (AOR: 3.11; 95% CI (1.15–8.36). Protective factor that could be intervened with was habit of taking a break (AOR: 0.44; 95% CI (0.3–0.8)). *Conclusion and Recommendation*. Three-fours of the employees were at risk. Sex, age, habit of taking a break, and use of electronic materials outside work environment were significantly associated with the presence of CVS. Remedial actions need to be considered at individual level.

## 1. Introduction

Computer technology has become the commonest and essential office tool. Unlike other office tools (telephone, written or printed page), its demand for vision is high. As a result, computer users may experience computer vision syndrome (CVS) [1, 2]. CVS is a significant public health problem and possible occupational epidemics of the 21^st^ century as it affects computer users from all walks of life [3–7]. An American Optometric Association has defined CVS “as a complex eye discomfort and vision problem such as eye strain (fatigue), blurred vision, excessive tearing, double vision, headache, light sensitivity, dry eye, and irritated eye experienced while using the computers for longer durations [8, 9].”

The health effects among users can be expressed in terms of stress, postures, health performance, and occupational productivity [10] and visual comfort [11]. This results in lowered productivity, increased error rate, reduced job satisfaction, and low quality of life [6, 12]. As revealed from research, the productivity can be reduced up to 40% [13].

Globally, a million new cases of CVS occur each year. According to the Seattle Times Company, worldwide, up to 70 million workers are at risk for computer vision syndrome, and these numbers are likely to grow. The report has indicated 70 percent to 90 percent of people uses computers extensively, whether for work or play, and have one or more symptoms of computer vision syndrome [14].

Currently in Ethiopia, in every institution computer is provided to all professional employees without considering the health effects resulting from using it [15]. As indicated by research, bank employees are at higher risk of eye problems compared to other employees [16]. Hence, the researcher expected a similar trend among the Ethiopian bank workers [13, 15]. A study done among bank employees before this work gives little attention in considering all personal factors, besides wearing eye glass, contrast and brightness of computer, and gender perceived benefits for CVS [13, 15, 17–19].

Therefore, the present study aims to describe the prevalence of CVS and its associated personal factors among employees of commercial Bank of Ethiopia. It fills the existing research gap via considering all personal factors and undertaking measurement. As a result, it has provided an evidence-based information to the little literature available on CVS in Ethiopia. The possible prevention methods of CVS generated from this study are helpful for ophthalmic professionals, health, and other training institutions in planning, developing, and revising training curricula that enhance knowledge and level of awareness regarding CVS among bank employees.

## 2. Methods

### 2.1. Study Area and Period

This study was conducted in the CBE situated at Addis Ababa, Ethiopia. The study was conducted from February 2018 to March 2018.

### 2.2. Study Design

An institution-based cross-sectional study was employed to assess the prevalence and personal factors of CVS among employees of commercial bank of Ethiopia in Addis Ababa.

### 2.3. Study Population

Source population were all employees of commercial bank of Ethiopia in all (four districts) who are computer users in Addis Ababa, Ethiopia. All bank employees who are working in the North and East Districts of Commercial Bank of Ethiopia were taken as study population.

### 2.4. Inclusion Criteria

The inclusion criteria included all bank workers who used computers to complete their tasks for at least 2 hours per day during the working days and had worked with the use of the computer for at least 12 months.

### 2.5. Exclusion Criteria

The exclusion criteria included bank workers who had eye problem due to other medical problems.

### 2.6. Sample Size Determination

The sample size for the prevalence of CVS among CBE workers was determined using a single population proportion formula with the following assumptions: level of significance (*α*) = 5% (at a confidence level of 95%), marginal error *d* = 5%, and *P*=0.73 (previous study on computer vision syndrome using bank workers at Gondar city) [15]. The *Z* value is 1.96 (*n* = sample size, *P* = proportion, and *d* = marginal error).

(1) $$\begin{array}{c} n={\left(\frac{z\alpha }{2}\right)}^{2}*p*\frac{\left(1-p\right)}{d^{2}},\\ n=\frac{{\left(1.96\right)}^{2}\times 0.73\times 0.27}{{\left(0.5\right)}^{2}},\\ n=303. \end{array}$$

By considering 10% nonresponses, the total sample size for prevalence of CVS was 333.

The sample size to determine personal factors associated with CVS among CBE employees was calculated using double population proportion formula in EPI INFO by taking three independent factors of CVS from the previous study. The maximum sample size was selected. Therefore, the sample size for this study was 359 (Table 1).

### 2.7. Sampling Technique

During the study period, in Ethiopia, there were 18 types of private and government banks. Considering its age, employee's level of exposure to computer, the number of employees and customers it has, and resource and time limitation, the researcher wants to conduct the study among employees of CBE in Addis Ababa [20].

**Figure 1:**
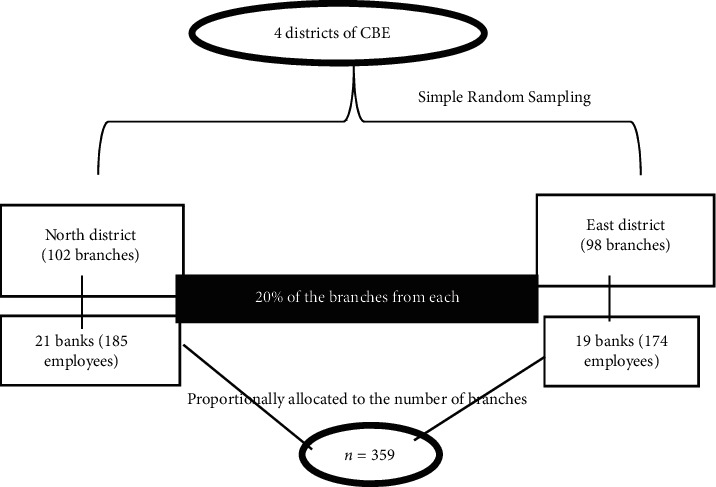
Schematic presentation of sampling procedure for the study of CVS among employees of commercial bank of Ethiopia, Addis Ababa, Ethiopia.

CBE has branches in four (4) districts (total of 400 branches), from which two districts (East and North district) were randomly selected by simple random sampling (lottery method) by considering the representativeness of the sample and logistics issues. With assumption of 20% of the branches, 40 banks were selected from both districts using lottery method and then the samples of study participants were allocated proportionally to the number of branches. Finally, the study participants of each bank were selected using simple random sampling (Figure 1).

### 2.8. Data Collection Tools

#### 2.8.1. Pretested Structured Questionnaire

Set of expert-validated self-administered questions were used to collect sociodemographic data, symptoms of CVS, and personal factors. Questions on symptoms of CVS were adapted from a previous study done by Ranasinghe et al. [18]. Presence of blurred vision, eye strain, eye fatigue, redness of eyes, watery eyes, eye dryness, double vision, eye irritation, burning sensation, and headache were assessed as symptoms of CVS. Presence of at least one symptom, either intermittently or continuously, during the previous 12 months in one or two eyes was considered as presence of CVS [18]. The questionnaire was prepared first in English and translated to Amharic (the national language of the study area) to make data collection process simple. To check its consistency in the meaning of words and concepts, the questionnaire was translated back to the English language by language experts after the data collection.

#### 2.8.2. Snellen Chart Measurement

This chart was used for testing distance vision or near blurred vision. The chart was placed on a wall 10 feet away from the participant. The participant covered one eye with his hand without applying pressure to the covered eye, as it might affect that eye's vision. The assistant (ophthalmic nurse) stands near the chart and record participants accuracy. The participant identifies a line on the chart which can be comfortably read and tried to read the letters on that line aloud. The participant continues trying to read the letters on each successively smaller line and have his/her assistant stop him/her when he/she fail to correctly identify at least 50 percent of the letters on a line. The participant switches his/her other eye and repeats the procedure again.

### 2.9. Quality Assurance of the Data

#### 2.9.1. Training of Data Collectors

Three data collectors, who are ophthalmic nurses, two BSc optometrist supervisors, and one environmental health professional, were recruited and given training for two days (one-day training and one-day field practice) on objectives of the study, the concepts of using the proposed sampling technique, eligibility criteria, structure of the data collection tool and the purpose of each item included in the tool, and techniques and ways of collecting the data as well as roles and responsibilities of the field team members.

Finally, having a brief discussion and taking corrective measures on the problems observed during the field practice, data collectors were ready to start the actual data collection. Therefore, data collection was begun at February. Two supervisors were also involved in monitoring and checking the completeness of the questionnaires.

#### 2.9.2. Data Collection Procedures

Informed written consents were taken from the study participants. By asking a permission from branch managers, the team accomplished the measurement using self-administered pretested questionnaires, eye segment examination using a Snellen chart, and direct observations of brightness and eye glasses. The self-administered questionnaires were filled by the participants at their homes and were received from the participants by the supervisor with roughly checking the questionnaires for the consistent responses after 24 hours. Hence, the data collection period has taken 20 working days.

#### 2.9.3. Data Processing and Analysis

After coding, the collected data were entered into a database using EPI INFO and were exported, cleaned (for any inconsistencies and missing values), and analyzed using SPSS version 21. Descriptive statistics (frequency, percentage) were used to describe main variables of interest.

Binary logistic regression analysis was done to see which variables are associated with the main outcome of interest. Multivariable logistic regression model was used to determine independent predictors of CVS.

#### 2.9.4. Ethical Consideration

Ethical approval was obtained from research ethics committee of Addis Ababa University. Official letters of cooperation from AAU and research center of CBE were written to respective study districts and branches. Informed consent was obtained from all study subjects to allow the use of anonymous personal and clinical data in research. Confidentiality of the information was maintained thoroughly by deidentification.

## 3. Results

### 3.1. Sociodemographic Characteristics

A total of three hundred fifty-nine (359) (216 male and 135 female respondents) employees of the CBE, Ethiopia, participated (response rate 98%). Majority of the study subjects 250 (71.2%) were in the age group range of 21–29 years old (Table 2).

### 3.2. Visual Acuity

The study reported that 343 (97.7%) have a right eye normal vision and 342 (97.4%) left eye normal vision while 8 (3.1%) have right eye low vision and 9 (2.6%) left eye low vision (Table 3).

### 3.3. Prevalence of CVS

**Figure 2:**
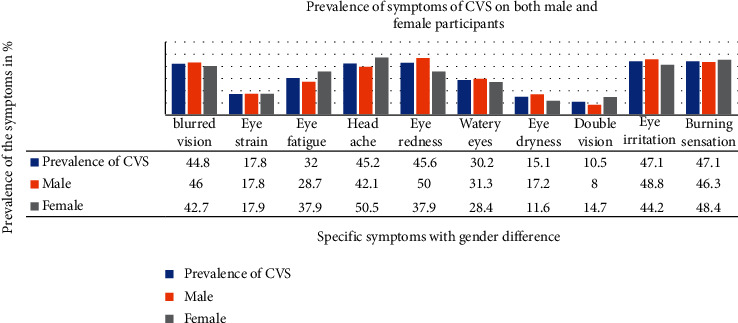
One-year prevalence of CVS symptoms among male and female commercial bank employees in Addis Ababa, Ethiopia, 2018.

The prevalence of CVS experienced in the last 12 months during computer use was 74.6% (95% [CI] = 70.1, 79.5) which was significantly greater in males (170, 64.9%) than in females (92, 35.1%) (Figure 2).

#### 3.3.1. Symptoms of CVS

**Figure 3:**
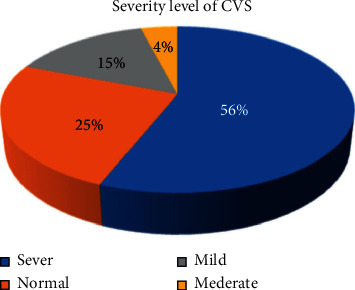
Symptoms of CVS among employees of Commercial Bank of Ethiopia in Addis Ababa, Ethiopia, 2018.

Severe symptoms of CVS were more prevalent among males (127, 65.1%) than females (68, 34.9%). From the study subjects who have CVS, 195 (74.4%) of subjects had severe symptoms. The overall severity status of CVS among the study subjects is as shown in Figure 3.

#### 3.3.2. Bivariate Logistic Regression

A binary logistic regression analysis was performed with “presence of CVS” as the dichotomous dependent variable and work experience (years), preexisting eye disease, habit of adjusting brightness and contrast of screen, age, sex, occupational difference, use of electronics out of work, having awareness, the habit of taking a break, and using computer eye glass, as the continuous/dichotomous independent variables.

Variables which showed association in the bivariate analysis at *P* < 0.2 were moved in to the multivariable logistic regression (Table 4).

#### 3.3.3. Multivariable Logistic Regression

Males were 1.8 times (AOR: 1.8; 95% CI (1–3)) more likely to develop CVS compared to females (Table 5).

## 4. Discussion

From this study, it has been observed that age, sex, use of electronics out of work, and habit of taking break are significantly associated with computer vision syndrome. A prevalence of 74.6% (95% confidence interval [CI] = 70.1, 79.5) was reported. The prevalence of CVS reported in this study is relatively similar to 74% [1], 73.9% [12], 73% [15], 72% [21], and 67.4% [18] in Nigeria, Ethiopia, UAE, and South Asian, respectively. The possible similarity to symptoms of CVS was considered.

On the contrary, a high prevalence of CVS was reported in Chennai among medical and engineering students (81.9%) [7], and in Malaysia [19] a prevalence of 89.9% was reported among university students. On the other hand, a lower prevalence of CVS (59.9%) was reported in Mauritius among keyboard users [22]. The reason for the high prevalence might be that participants in other studies might have had comfortable situations to take a break and blink eyes. As shown above, the high prevalence is among university students who might be at higher tension to read, write, and prepare projects in different workplaces proximal to environmental factors and they might use other electronics materials other than computer such as tablets and mobiles for educational and recreational purpose. However, bank workers usually take rest during customer services.

On the other hand, the definitions for CVS are not consistent. For example, the studies in Chennai and Malaysia include musculoskeletal symptoms such as neck and shoulder pain as additional symptoms of CVS, whereas the definition of CVS in this study consisted only of eye/visual symptoms. The other reason might be the exposure duration used. The study in China used an exposure duration of one month which might not be exposed to recalling bias relative to the current study (take the past 12 months as exposure duration). The reason for the lowest prevalence might be due to the lowest response rate. A questionnaire-based survey which was carried out among 362 computer users in Mauritius resulted in the fact that 200 (62%) completed the questionnaire [22].

In many studies, it was observed that the proportion of females who developed CVS was more compared to the prevalence among males [18, 22–24]. However, in this study being male was significantly associated with the risk of developing CVS. This is supported by Logaraj et al. who reported that individual symptoms such as redness, burning sensation, blurred vision, and dry eyes were comparatively more in males than in females [7]. The reason why CVS is common in males in this study might be their exposure status outside work compared to those females who might be at work other than using computer.

On the other hand, in this study workers who are users of electronic materials (computer, tablet, and smart phone screen) outside work in addition to their work area were three times more likely to suffer from CVS as compared to those who are using electronic materials at work area only. This is possibly due to using the electronics in dark places and high exposure time for educational and recreational purpose. Likewise, study participants in the age group of 30–39 years were 3 times more likely to suffer from CVS as compared to those who are in the age group of 20–29 years. Workers at the age group of more than 40 years were not associated with CVS. This is possibly due to the small number of study participants in the age group of more than 40 years.

Participants who have the habit of taking a break were 56% less likely to develop CVS compared to those who do not have the habit of taking a break. In the same fashion, studies showed that significant correlation was found between taking less frequent breaks or working on computer for more than 20 minutes without break and being more exposed for symptoms of CVS when compared with taking more frequent breaks within 20 minutes [7, 15, 17, 23, 25, 26]. But in this study it was difficult to look for associations between the specific time of taking a break and CVS, since the participants' habits of taking a break were different. The reason why the habit of taking a break is preventive is that it is related to relaxing the muscles of the eyes which can then decrease the eye fatigue and headache [27]. However, other studies demonstrated that taking a break was not significantly associated with prevalence of CVS [18, 19]. This may be due to the difference in giving definition for the habit of taking a break. Those studies who came up with association of taking a break and prevalence of CVS use definition for habit of taking break: taking 15-minute break of 1–2 hours interval and refreshing the eyes every 20 minutes while at computer at use. Using eye glass was also associated but the confidence interval and odds ratio were too much high which is difficult to believe due to small numbers of participants.

## 5. Limitation of the Study

As this study was a cross-sectional study and used self-administered questionnaire, social desirability bias and recall bias might possibly be introduced. Individuals who are severely sick and absent from work might not be included during the data collection. Most of the eye symptoms were self-reported except blurred vision. Future researchers have to address eye symptoms using medical examination.

## 6. Conclusions

This study reported a high prevalence (74.6%) (95% confidence interval [CI] = 70.1, 79.5) of computer vision syndrome with the majority (56%) having severe cases. Sex, age, habit of taking a break, and use of electronics outside work environment were significantly associated with CVS. The problems can be managed through continuous training.

## Figures and Tables

**Table 1:** Sample size to determine personal factors associated with CVS among bank workers in Addis Ababa, Ethiopia, 2018.

Independent factors at multivariable analysis	Exposed	Nonexposed	OR	Sample size	10% nonresponders
Sitting position	67.17	83.9	2.547	230	253
Taking break	62.5	77.3	2.04	326	359
Eye glass	71	88.57	3.16	184	202

**Table 2:** Sociodemographic characteristics of computer user bank workers in Addis Ababa, Ethiopia, 2018 (*n* = 359).

Variables	Categories	Frequency	Percent (%)
Age	20–29 years	250	71.2
	30–39 years	80	22.8
	≥40 years	21	6

Work experience	<5 years	224	63.8
	≥5 years	127	36.2

Job title	Managerial and administrative	63	17.9
	Clerical and professional	288	82.1

Working hours per day	3–7 h	124	35.3
	Above 7 h	227	64.7

Sex	Female	135	38.5
	Male	216	61.5

**Table 3:** Visual acuity distribution among employees of Commercial Bank of Ethiopia, 2018.

Categories	Male		Female		Total	
	Right eye	Left eye	Right eye	Left eye	Right eye	Left eye
Normal (6/6–6/18)	212	212	131	130	343	342
Low vision (6/18–6/60)	4	4	4	5	8	9

**Table 4:** Bivariate analysis for computer vision syndrome among bank workers in Addis Ababa, Ethiopia, 2018, *n* = 359.

Variables	Categories	Computer vision syndrome		COR (95% CI)	*P* value
		Yes (%)	No (%)		
Age	≤29	171 (65.3)	79 (88.8)	1	
	30–39	72 (27.5)	8 (9.0)	4.16 (1.9–9.04)	0.000 (<0.001)^*∗*^
	≥40	19 (7.3)	2 (2.2)	4.39 (0.99–19.38)	0.05^*∗*^

Sex	Female	92 (35.1)	43 (48.3)	1	
	Male	170 (64.9)	46 (51.7)	1.73 (1.06–2.8)	0.028^*∗*^

Work experience	<5 years	138 (52.7)	86 (96.6)	1	
	≥5 years	124 (47.3)	3 (3.4)	25.7 (8–84)	0.000 (<0.001)^*∗*^

Job title	Managerial	54 (20.6)	9 (10.1)	1	
	Others	208 (79.4)	80 (89.9)	2.31 (1.1–4.89)	0.029^*∗*^

Awareness	No	122 (46.6)	42 (47.2)	1	
	Yes	140 (53.4)	47 (52.8)	1.025 (0.63–1.67)	0.919

Use of electronics out of work	No	15 (5.7)	11 (12.4)	1	
	Yes	247 (94.3)	78 (87.6)	2.322 (1.024–5.265)	0.044^*∗*^

Habit of taking break	No	183 (69.8)	50 (56.2)	1	
	Yes	79 (30.2)	39 (43.8)	0.55 (0.337–0.908)	0.019^*∗*^

Using eye glass	No	223 (85.1)	88 (98.9)	1	
	Yes	39 (14.9)	1 (1.1)	15.4 (2–114)	0.007^*∗*^

Preexisting disease	No	244 (93.5)	78 (87.6)	1	
	Yes	17 (6.5)	11 (12.4)	0.494 (0.222–1.1)	0.084

Habit of brightness and contrast adjustment	No	120 (45.8)	42 (47.2)	1	
	Yes	142 (54.2)	47 (52.8)	1.057 (0.653–1.712)	0.820

^*∗*^Variables which were significant in the first model, 1: reference, COR: crude odds ratio, CI: confidence interval.

**Table 5:** Multivariate analysis for computer vision syndrome among bank workers in Addis Ababa, Ethiopia, 2018, *n* = 359.

Variables	Categories	Computer vision syndrome status		COR (95% CI)	AOR (95% CI)	*P* value
		Yes (%)	No (%)			
Age	≤29	171 (65.3)	79 (88.8)	1	1	
	30–39	72 (27.5)	8 (9.0)	4.16 (1.9–9.0)	3.1 (1.2–8)	0.022^*∗*^
	≥40	19 (7.3)	2 (2.2)	4.39 (1–19.4)	3.4 (0.6–18.4)	0.159

Sex	Female	92 (35.1)	43 (48.3)	1	1	
	Male	170 (64.9)	46 (51.7)	1.7 (1.1–2.8)	1.8 (1.0–3.0)	0.039^*∗*^

Use of electronics out of work	No	15 (5.7)	11 (12.4)	1	1	
	Yes	247 (94.3)	78 (87.6)	2.3 (1.0–5.3)	3.11 (1.2–8.4)	0.025^*∗*^

Habit of taking break	No	183 (69.8)	50 (56.2)	1	1	
	Yes	79 (30.2)	39 (43.8)	0.55(0.34–0.9)	0.44(0.3–0.8)	0.004^*∗*^

^*∗*^Variables which were significant in the final model, 1: reference, AOR: adjusted odds ratio, CI: confidence interval.

## Data Availability

The data will be made accessible from the primary author upon request.
